# Recovery trajectories of kelp forest animals are rapid yet spatially variable across a network of temperate marine protected areas

**DOI:** 10.1038/srep14102

**Published:** 2015-09-16

**Authors:** Jennifer E. Caselle, Andrew Rassweiler, Scott L. Hamilton, Robert R. Warner

**Affiliations:** 1 Marine Science Institute, University of California, Santa Barbara, CA 93106 USA; 2 Moss Landing Marine Laboratories, 8272 Moss Landing Rd., Moss Landing, CA 95039 USA; 3 Department of Ecology, Evolution, and Marine Biology, University of California, Santa Barbara, CA 93106 USA

## Abstract

Oceans currently face a variety of threats, requiring ecosystem-based approaches to management such as networks of marine protected areas (MPAs). We evaluated changes in fish biomass on temperate rocky reefs over the decade following implementation of a network of MPAs in the northern Channel Islands, California. We found that the biomass of targeted (i.e. fished) species has increased consistently inside all MPAs in the network, with an effect of geography on the strength of the response. More interesting, biomass of targeted fish species also increased outside MPAs, although only 27% as rapidly as in the protected areas, indicating that redistribution of fishing effort has not severely affected unprotected populations. Whether the increase outside of MPAs is due to changes in fishing pressure, fisheries management actions, adult spillover, favorable environmental conditions, or a combination of all four remains unknown. We evaluated methods of controlling for biogeographic or environmental variation across networks of protected areas and found similar performance of models incorporating empirical sea surface temperature versus a simple geographic blocking term based on assemblage structure. The patterns observed are promising indicators of the success of this network, but more work is needed to understand how ecological and physical contexts affect MPA performance.

Globally, oceans are facing a large number of threats including overfishing, pollution, eutrophication, sedimentation, and climate change. There are no areas left in the oceans that are unaffected by humans^1^. These human-induced changes are impairing the ocean’s capacity to provide food, protect livelihoods, maintain water quality, and recover from environmental stress. These and other benefits, collectively called “ecosystem services”, depend on ocean health^2^. The scale of most human impacts to the ocean goes beyond single habitats or species and as such, requires a more holistic, ecosystem-based approach to management^3^. Marine protected areas (MPAs) are a commonly implemented approach for conserving biodiversity and managing marine resources. By protecting populations, habitats, and ecosystems within their borders, MPAs can provide a spatial refuge for the entire ecological system they contain. MPAs can also provide a powerful buffer against a naturally fluctuating environment, catastrophes such as hurricanes, and the uncertainty inherent in traditional marine management actions^4^.

Considerable scientific research shows that MPAs and marine reserves (defined as no-take MPAs) increase the biomass, abundance, diversity, and size of marine species living within their borders (for review see^5^), with stronger effects in the no-take marine reserves^6^,^7^,^8^. Generally, species that are subject to fishing pressure outside MPAs show the greatest increases in response to protection while other species may show no response, or even decline^9^,^10^,^11^,^12^,^13^,^14^. Such declines may reflect interactions among species, where larger and more abundant predators inside MPAs (often those species most prone to overfishing) have cascading effects on lower trophic levels^15^,^16^. For example, in New Zealand, overfishing of a predatory lobster outside of MPAs reduced the resilience of kelp beds to climate-induced shifts towards urchin barrens^15^. In the California Channel Islands, the buildup of two sea urchin predators (California sheephead, a fish, and California spiny lobster) inside a long-standing, fully protected marine reserve resulted in a decline in sea urchin abundance and a subsequent increase in kelp^16^,^17^. In addition to trophic interactions, the effects of habitat heterogeneity and environmental conditions can also lead to unpredictable responses of organisms inside MPAs and must be taken into account when assessing the effects of spatial protection measures^18^,^19^,^20^,^21^. Finally, the amount and spatial distribution of fishing will influence the responses seen in and around MPAs. Both models^22^,^23^,^24^ and empirical evidence^25^,^26^ have suggested that redistribution of fishing effort outside MPAs, in particular ‘fishing-the-line’ behavior, may lead to declines in species abundance or delays in recovery.

Marine protected area size is a key determinant of success. By themselves, small MPAs may not support populations that are large enough to sustain themselves or sustain fisheries in the adjacent open areas^6^. Additionally, small MPAs may not contain a sufficiently high diversity of important habitats or species to meet biodiversity goals. While recently there has been a trend towards implementation of very large protected areas (e.g. Phoenix Islands Protected Area, Pacific Remote Islands Marine National Monument, and Chagos Marine Protected Area), in many regions economic constraints make it impractical to create a such large reserves^27^. An alternative approach is to establish networks of several smaller MPAs, which may help reduce localized economic impacts without compromising conservation and fisheries benefits^28^,^29^,^30^. A network generally includes a set of multiple MPAs, located in critical habitats, and designed to be connected by the dispersal of larvae and/or movement of juveniles and adults^31^. In an effective network, organisms must be able to travel beyond the boundaries of a single protected area into other protected areas. By using different sizes and spacing of protected areas, a network can protect species with different life history and behavioral characteristics, and may offer a better compromise between human use and conservation than single large protected areas.

For all the potential benefits of well-designed MPA networks, they pose many difficulties in assessing MPA performance^31^. Often, and even by definition, a network is placed across a biogeographic region and designed to capture a variety of habitat types and environmental characteristics^32^,^33^. While this may be useful for protecting a wide range of species, assessment is challenging because the effect of each MPA in the network may be different, depending on the traits and life-histories of the species it contains, the variety of environmental characteristics it experiences, and the spatial distribution of human usage around and within it.

Another challenge in assessment of any MPA, whether contained within a network or not, is the difficulty in separating natural spatial variation from the effects of protection. This problem may be particularly pronounced if MPAs are placed non-randomly, for example if placed in locations that are either particularly rich or poor in biodiversity or biomass, or targeted to contain certain habitats. Such non-random placement can be the result of strategic design or political constraints. One solution to this problem is to implement an analytical design that includes data comparing communities inside and outside of protected areas, both before and after protection (i.e., Before-After-Control-Impact or ‘BACI’ design^34^,^35^). Unfortunately it is quite rare that suitable data exist from before MPA implementation (but see^36^,^37^). Here we explore a variant of the more commonly used control-impact approach: we compare trends over time inside and outside of protected areas after implementation^35^. By focusing on change over time, we can examine the effects of protection in a way that is much less susceptible to simple biases in MPA placement, such as MPAs being placed in locations with more or fewer fish. We note however, that this approach would not correct for more subtle biases such as MPAs being placed in locations where fish populations are increasing or expected to increase over time.

Regardless of the basic statistical design, another significant and underexplored problem remains when comparing protected areas to reference areas (i.e., controls): MPAs are expected to and are often explicitly designed to affect populations outside their boundaries. This effect may be positive, through the export or spillover of MPA production and the movement of larvae or adults to fished areas^38^,^39^; or it may be negative, due to displaced fishing effort increasing pressure in non-protected areas^25^,^26^. Thus a simple comparison of conditions inside versus outside of MPAs, such as the commonly used response ratios, may mask actual deterioration or improvement in ‘control’ areas^40^. The temporal data that are at the heart of our analyses can address this problem directly by examining the trajectories of change in MPAs and nearby fished areas. When a network design allows these comparisons to be extended geographically across biogeographic regimes and gradients of fishing pressure, we can begin to identify the critical factors affecting the performance of MPAs. Previous work has implied that choosing control locations that are likely to be affected by the MPA should be avoided and that areas outside of the influence of the MPA would be better controls^35^. However, when MPAs are placed in extensive regional networks, there may be no areas that are not influenced by a MPA.

Here we investigate patterns of temporal change in temperate kelp forest fish communities over a ten-year period across a network of temperate MPAs in the Santa Barbara Channel, California. In 2003, eleven MPAs (9 no-take marine reserves and 2 partial-take MPAs) were placed across the northern Channel Islands^32^. This network encompasses a large range in environmental variation, with cold water in the west blending with warmer water in the east (Fig. 1), substantial variation in productivity^41^, and resulting biogeographic variation in community structure^9^,^42^. Previously, we showed the importance of controlling for these biogeographic differences when assessing overall network performance, and evaluated biological responses in the aggregate over the first five years post-implementation^9^. Here we extend that work by showing rapid and sustained change across the network over a decade since implementation, both inside and outside of MPAs situated on four islands (Fig. 1). We also evaluate several methods of controlling for biogeographic variation in analyses that test for the effects of MPAs on patterns of fish biomass. We conclude with a discussion of factors responsible for determining the timing of responses in MPAs and suggestions for measuring those effects.

## Results

**Figure 1:**
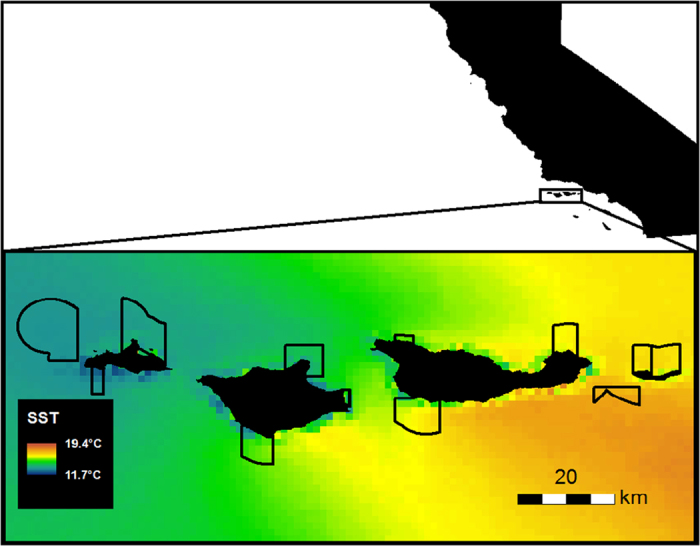
Map of the northern Channel Islands showing Marine Protected Area boundaries and average of sea surface temperature (SST) calculated from 15-day means over the period 2000-2012. SST data were MODIST (2000-present) and MODISA (2002-present) with 1km resolution (see methods). Map was created by the authors using ArcGIS 10.

**Figure 2:**
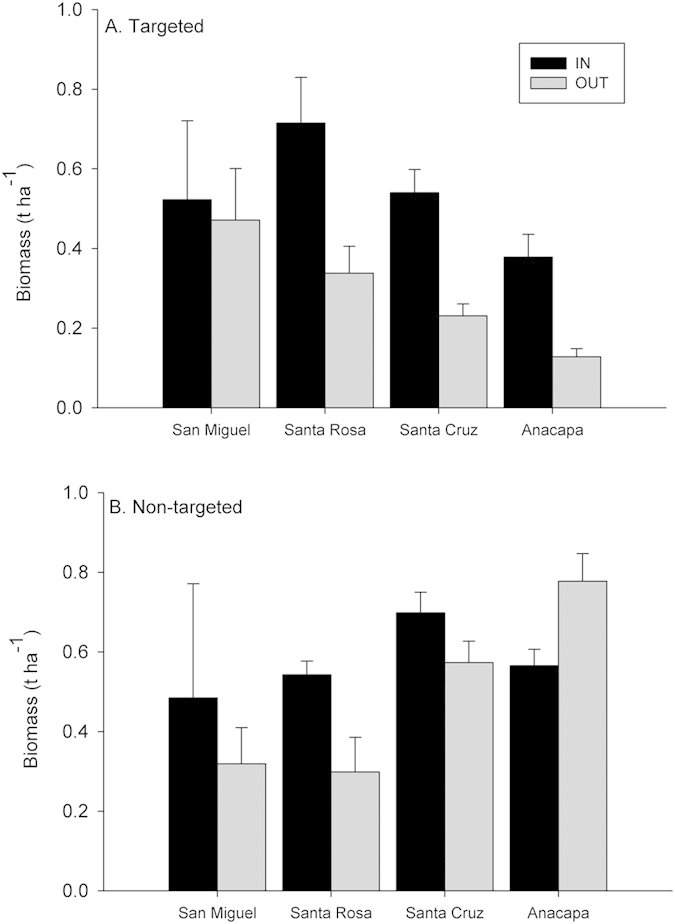
Spatial variation in total fish biomass (metric tons per hectare) inside (black) and outside (grey) MPAs across the network presented by island. (**A**) Average total biomass of all fish species targeted by fishing. (**B**) Average total biomass of all fish species not targeted by fishing. Means for years 2005–2012 +/− 1 SE

As predicted by marine reserve theory, we found that the total biomass (averaged over 2003–2012) of fish species targeted by fishing was greater inside of MPAs, where fishing is prohibited, than outside of MPAs, where fishing is allowed, for all 4 northern Channel Islands (Table 1A and Fig. 2A). We also detected an additional significant effect of island; fish biomass was highest at the western islands. For targeted species, three of the four islands showed a large MPA effect. Although there was no significant interaction between island and protection, the MPA on San Miguel Island, the furthest west from port, most exposed, and coldest island (Fig. 1) had the most similar biomass to its reference site. By contrast, for species not targeted by fishing, there was no consistent pattern of response to protection (Table 1B). There was a significant effect of island on biomass (Fig. 2B), with biomass being highest at the eastern islands for non-targeted species.

**Figure 3:**
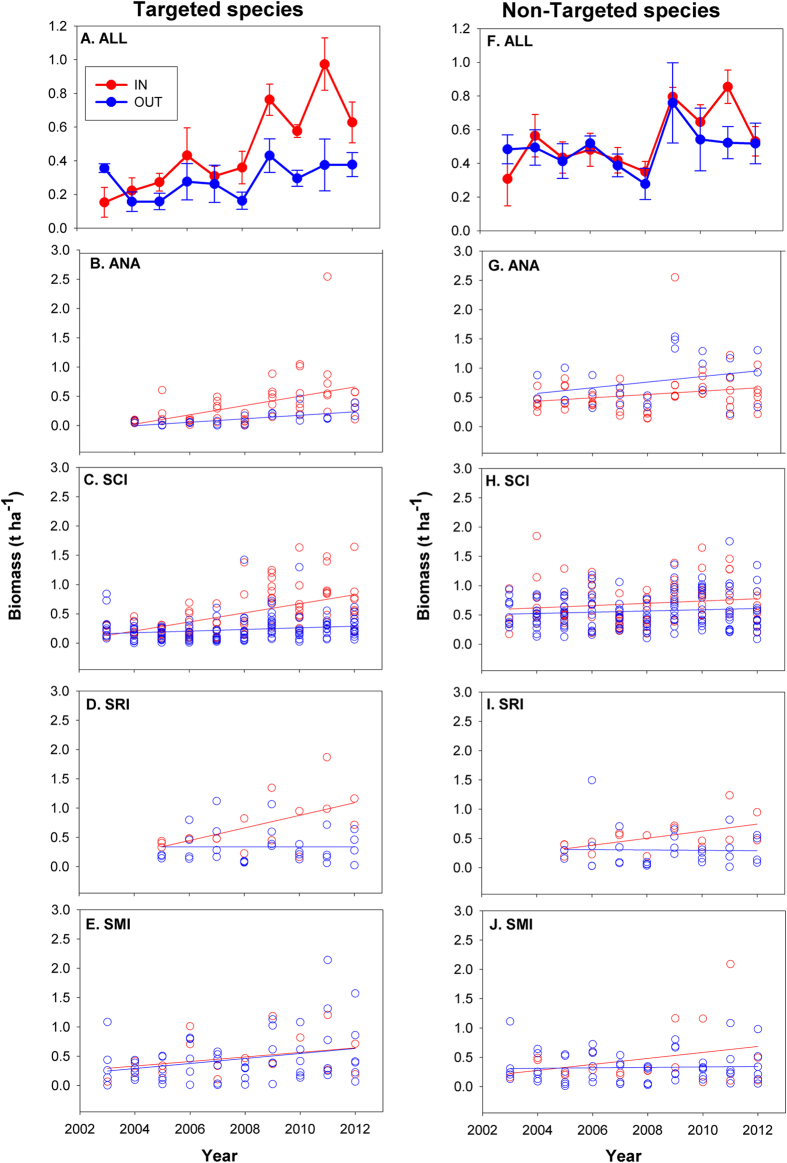
Trajectories of change in biomass (metric tons per hectare) inside (red) and outside (blue) MPAs across the islands in the Northern Channel Islands for targeted (left panels) and non-targeted (right panels) fish species. Panels A and F show mean total biomass across all regions since MPA establishment in 2003. Panels B-E and G-J present biomass trends for each island across the network from East (Anacapa) to West (San Miguel).

Underlying the spatial variation in average biomass as a function of protection status, we found substantial differences in the trajectories of change in fish biomass over time between MPAs and unprotected sites. We present these temporal data first in the aggregate (Fig. 3A,F), and then group sites by island, using simple linear regression to illustrate the relative trends on each island for MPA and non-MPA sites (Fig. 3). Biomass of targeted species increased significantly faster inside MPAs than outside of MPAs (~4× faster; Fig. 3A), as evidenced by the highly significant positive interaction between protected status and time. This interaction was present both in statistical models using island as a categorical variable (Table 2A), and those using mean SST at each site as a continuous covariate to control for east-west environmental gradients (Table 3A). The strong and significant interaction in both models means that the main effects for protected status and time cannot be interpreted separately. There was also a significant effect of biogeography on the response of targeted species to MPA protection for both models (Island: Table 2A; mean SST: Table 3A). While targeted fish species biomass tended to increase everywhere from 2003–2012, the rate of change, while not statistically significant (i.e. non-significant island*year*protection and island*year effects), appeared to be faster inside than outside MPAs on the warmer, eastern islands, that are closer to port, than the more distant, cooler, and more exposed westernmost island (San Miguel) (Fig. 3B–E). At San Miguel Island, the rate of change in biomass was more similar inside and outside of MPAs (Fig. 3E).

In contrast, the biomass trajectories of non-targeted fish species did not show significantly different trends inside vs. outside of MPAs (i.e. an interaction between protected status and time; Fig. 3F and Tables 2B and 3B). The biomass of non-targeted fish tended to increase over time in both the model including island as a categorical variable (Table 2B) and in the model including SST (Table 3B). This effect was primarily driven by an increase in biomass in 2009 at several locations inside and outside the MPAs, which may be partially explained by favorable recruitment for many species during the year or two prior. The biogeographic effect was significant in both models (Tables 2B and 3B and Fig. 3G–J) but the results were complex. Biomass increased fastest outside MPAs at the warmest island (Fig. 3G) and inside MPAs at the two coldest islands (Fig. 3I,J). As expected in this dynamic ecosystem, there was a high variance among locations both inside and outside MPAs.

### Model fits

We tested the effect of using satellite-derived long-term averages of sea surface temperature at the sites rather than categorical island-based blocking (from^9^ in the linear models presented above; Supplementary Table S1). For targeted species, both representations of biogeography were highly significant and the overall model fit was very similar (Table S1A; r^2^ is 0.33 and AICc is 728.78 for the model with island blocking while r^2^ is 0.32 and AICc is 720.65 for the model in which SST is used to account for biogeography). For non-targeted species, again both SST and island were highly significant predictors of biomass but in this case, island-based blocking substantially outperformed SST as a biogeographic covariate (Table S1B; AICc is 663.00 for the model with island blocking while AICc is 698.61 for the model in which SST is used to account for biogeography). However, both non-targeted species models had low fit overall (r^2^ = 0.20 and 0.13 for the models with island blocking and SST respectively).

### Changes in fish biomass outside of MPAS

To understand fishing-related changes in biomass in unprotected areas (due to MPA effects, redistribution of fishers, and/or changes in management), we compared the trajectories of biomass of targeted and non-targeted species outside of MPAs only (i.e. the outside trajectories from Fig. 3A,F). We found a significant effect of targeted status (F_1,514_ = 60.7, P < 0.0001) and year (F_1,514_ = 14.7, P = 0.0001) as well as a significant interaction between year and targeted status (F_1,514_ = 5.5, P = 0.02) and between island and targeted status (F_3,514_ = 37.9, P < 0.0001), indicating that while biomass increased outside of MPAs for both groups of species, there was no strong effect of island on that pattern. In general, we note that in no island*targeted-status grouping did biomass significantly decline outside of MPAs.

### Site-specific fish biomass trajectories

**Figure 4:**
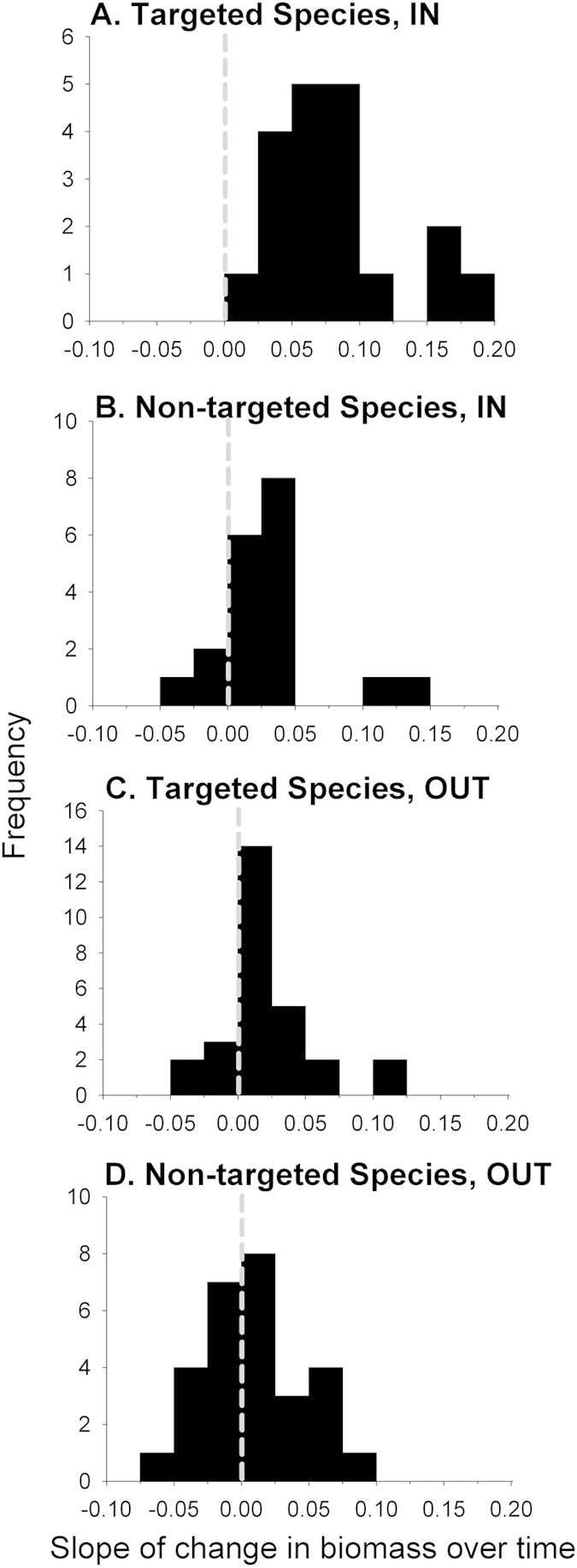
Frequency histograms of the rate of change in fish at individual sites in the Channel Islands. Values are estimates of the annual change in biomass (t ha^−1^) calculated as the slope of the relationship between biomass and time since MPA establishment. Shown are histograms for sites inside (**A**,**C**) and outside (**B**,**D**) MPAs for targeted (**A**,**B**) and non-targeted (**C**,**D**) fish species. Positive slopes represent increasing biomass and negative slopes indicate decreasing biomass over time. Vertical dashed line at zero represents no change in biomass.

We calculated the rate of change over time (i.e., the slope of the trend in biomass from 2003–2012) at each site for the fish species groupings (targeted vs. non-targeted) and plotted the distribution of positive and negative slopes by protection level and targeted status (Fig. 4). At every within-MPA site sampled, total biomass of targeted species increased over time (the slope is positive for 19 out of 19 MPA sites, Fig. 4). The mean slope is 0.078 t ha^−1^ yr^−1^ and is significantly different from zero (t_18_ = 7.70, p < 0.0001). Importantly, total biomass of targeted species also increased *outside* of MPAs, at 23 out of 28 non-MPA sites with an average slope of 0.021 t ha^−1^ yr^−1^ (t_27_ = 3.09, p = 0.0046). Total biomass of non-targeted species also increased at the vast majority of within-MPA sites (18 out of 21 sites). The average rate of change was lower than that observed for species targeted by fishing activities (0.028 t ha^−1^ yr^−1^); however, the mean slope was still significantly different from zero (t_18_ = 3.21, p = 0.0048). In contrast, outside of MPAs the distribution of slopes of site-specific changes in biomass of non-targeted species did not differ significantly from zero (t_27_ = 1.50, p = 0.1445) and the average slope was 0.01 t ha^−1^ yr^−1^, indicating that on average this group neither increased nor decreased in biomass over the time period studied.

### Species-specific fish biomass trends

The trajectories of change in aggregate (total) biomass of non-targeted and targeted species groupings are mirrored in the changes in biomass of individual species or genera (Supplementary Table S2). Many of the targeted species had significant, positive slopes inside MPAs and the results for models using island and SST as blocking terms were similar (Supplementary Table S2A and B, respectively). Two heavily fished species in the region, California sheephead and Kelp Bass, both showed clear patterns of increasing biomass inside MPAs and no change outside. Trends for the non-targeted species were less clear; species showed increases and decreases both inside and outside of MPAs.

## Discussion

Beginning in 2007, under the auspices of the Marine Life Protection Act (MLPA), the State of California implemented the second largest MPA network in the world^43^,^44^,^45^. Included in this statewide network was a series of protected areas in the northern Channel Islands that were established considerably earlier, in 2003, following a separate process. While the Channel Islands MPAs span a much smaller area than entire MLPA network, they are located across the boundaries of two major biogeographic regions^32^. The state of California mandated a review of the status of the northern Channel Islands MPA network five years after its implementation. At that time, we documented the extreme variation in kelp forest community structure across the MPA network and showed that accounting for that variation in analyses provided more power to detect MPA effects than models that did not account for geography^9^. The review was a snapshot in time, evaluating the differences between protected and unprotected sites averaged over the five-year period.

Here we expand on those results by investigating in detail the trajectories of change across the network over a decade, asking whether the underlying geographical differences in community structure or environmental conditions (i.e., SST) lead to differences in the rate of response to protection over time. In addition, by investigating the trajectories of change in areas outside of MPAs, we ask whether there is any evidence that MPA establishment has led to positive changes outside (perhaps through export of production from inside) or to negative changes (perhaps due to displaced fishing effort)^46^. It is worth noting that there is no evidence that MPAs were originally placed in either degraded or richer areas compared to non-MPA sites: In every case, the biomass values at the initiation of this study inside MPAs are no different from those outside MPAs (Fig. 3). Further, our previous work showed no consistent differences in habitat among the protected and unprotected sites within each island^9^, although strong habitat differences do exist among islands.

Our analyses focus on differences in response between groupings of targeted (i.e., fished or exploited) and non-targeted (i.e., unfished, unexploited, or incidentally caught) fish species, inside vs. outside of MPAs. We hypothesized that by eliminating fishing pressure, the earliest effects of MPAs should be greatest at protected sites and on those species that are fished^13^. Just as we explicitly allow for the possibility that the existence of MPAs can cause changes in areas without protection, we expect MPAs to affect non-targeted species within their borders through trophic interactions (e.g., buildup of predators affecting prey species in MPAs) or competition^11^,^12^,^47^. Thus changes through time for non-targeted species inside MPAs are to be expected and will be highly informative. However, these indirect effects may take longer to manifest, and can be difficult to detect in highly dynamic temperate ecosystems^13^,^48^. Further, detecting these indirect effects will likely require species-specific analyses, targeting known trophic or competitive pathways among species, such as the well-known urchin predator (lobster or fish), urchin, and kelp interaction^15^,^16^. Here we simply test for effects of protection on total biomass of fished versus unfished species groups; future analyses of individual species with trophic or ecological linkages will allow inference of more complex effects of MPAs over time^16^.

Temperate kelp forest communities are well known to change dramatically in relation to environmental conditions, including ‘phase shifts’ or changes in dominance from one set of organisms to another^49^,^50^, potentially obscuring changes due to human management interventions. While we did observe large interannual variability, the trajectories of change in biomass for targeted fish species (in the aggregate and for individual species) were positive inside MPAs across the Channel Islands region, continuing the pattern observed in the 5-year review^51^. One should not expect this pattern of increase to be maintained forever, of course. At some point, the protected species should reach a level of biomass set by the carrying capacity of the protected area^52^,^53^; at that time the trajectories of biomass will primarily reflect year-to-year changes in environmental or ecological conditions. For example, in a nearly 40 year study of reserves in Kenya, it was shown that the time course of recovery to equilibrium biomass depended on a species’ life history and biological interactions (e.g. competition, predation, etc.)^52^. Some species increased rapidly, only to decrease in abundance as the successional process continued. While some studies have indicated that it might take multiple decades to detect changes in marine reserves depending on the life history traits (e.g., growth rate, reproductive output, mobility, etc.) of the species involved^54^, others have reported that in temperate^13^ and tropical^55^ reserves a positive response of targeted species to reserve protection can be quite rapid and occur over large spatial scales.

In contrast to the biomass trajectories of targeted species, non-targeted species in our study showed no such consistent pattern of change over time or difference between sites inside and outside of MPAs, either in the aggregate (Fig. 3F) or when broken down into geographic areas (Fig. 3G–J). This is not surprising because this grouping of species included fishes with a broad range of body sizes, trophic groups, and life history characteristics. The most striking trend in the data was a sharp increase in biomass in 2009, which we believe was driven by a large multi-species recruitment event that occurred at a few sites located both inside and outside of MPAs, possibly over several years.

The rate of change of targeted species outside of marine reserves is a combination of four effects: (1) export from the reserves (a positive effect), (2) depletion from fishing, including displaced fishing (a negative effect), (3) fisheries management regulations outside of MPAs, and (4) general environmental effects (positive or negative, which may be reflected by changes in non-targeted species). In practice, it is difficult to disentangle these effects, but one thing is clear from the trajectories shown in Fig. 3: there is no evidence that targeted fish species are being depleted outside of MPAs in the Channel Islands due to displaced fishing effort, which is a commonly cited criticism of MPAs. On average, the rate of change of targeted species inside MPAs was 4x that of targeted species outside MPAs but both slopes were positive. As well, at individual sites across the study region there are proportionally more instances of positive changes over time in targeted species outside of MPAs than seen in non-targeted species, which were predicted not to change in response to MPAs (compare Fig. 4C with 4D). In the Channel Islands, like many regions of the world, we do not have data on the spatial distribution of fishing at appropriate scales needed to address the changes we observed outside MPAs. Thus, we cannot address the possibility that there was an overall reduction in fishing effort in the region due to loss of fishermen from the industry, nor can we address the extent, if any, of spillover to adjacent unprotected areas. Studies of the California spiny lobster in the Channel Islands did show evidence of rapid buildup of density and subsequent spillover from several MPAS^20^, and previous studies of movement patterns of the same fish species found in these MPAs indicate the potential for spillover^56^. Detailed spatial patterns of abundance of fished species relative to MPA boundaries and fishery activities have been the subject of several works in the past^57^,^58^, and are an important subject of continuing work in the Channel Islands.

Many factors influence response rates of populations inside and outside MPAs. Abesamis *et al.*^54^ reviewed decades of research on rates of change of coral reef fishes that were categorized by life history characteristics such as body size, growth rates, life spans, and age and size at maturity^59^,^60^. They hypothesized that changes resulting from marine reserve protection should take longest for species that are large, slow-growing, and long-lived. In addition, Claudet *et al.*^11^ showed that for commercially exploited fish species, life history traits such as body size, adult habitat, mobility, and behavior had strong effects on the response to protection, while unexploited species showed little response to protection or relevance of life history and ecological traits. Modeling studies also suggest that population responses to protection are related to the generation time of the species of interest, as well as fishing pressure and reserve size^46^. The longer the generation time (i.e., slow growing, long-lived), the longer the response time. In the Channel Islands, like many regions of the world, the traits that correlate with slower responses (e.g. long life span, slow growth, large size, older age at maturity) tend to characterize more highly targeted, piscivorous or carnivorous species such as rockfishes (genus *Sebastes*) or California sheephead (*Semicossyphus pulcher*). These species are predicted to respond more slowly, but we saw rather rapid responses. This may be because we grouped all ‘targeted’ fishes, and so included both the longer-lived, heavily fished piscivores as well as a number of more lightly fished omnivores and planktivores that would be expected to respond more rapidly. Again, future species-specific analyses may detect variation among the targeted fish species in rates of change in relation to variability in life-history and behavioral traits (see Table S2).

We previously demonstrated the importance of biogeography in explaining MPA responses across the strong environmental gradient in the Channel Islands^9^. The most dramatic biogeographic patterns align with sea surface temperature gradients (Fig. 1), although other factors such as productivity, wave exposure, and kelp species diversity and persistence also vary along the gradient of SST^41^,^42^,^61^. Strong and persistent differences in these environmental forcing factors are associated with kelp forest communities possessing different characteristics, but these regions also may differ in the amount of fishing pressure due to distance from port and levels of exposure to wind and waves^62^. The most striking difference seen in responses to protection is at San Miguel Island, which is westernmost, coldest, and farthest from port (Fig. 1). In MPAs, targeted species biomass appeared to increase slightly over time in this region, but the pattern did not differ from that seen in areas without protection (Fig. 3E), nor did it differ from the pattern seen in non-targeted species (Fig. 3J). The community of targeted fish species at San Miguel is comprised primarily of slow-growing, long-lived rockfish species. We hypothesize that these species traits, combined with reduced and highly intermittent recruitment in these colder regions^63^ leads to a slower response to protection. Another, non-mutually exclusive hypothesis for lack of reserve effect is that fishing pressure on the fish species studied here was not intense prior to protection (and thus responses to protection will be small or nonexistent). In this study, we monitor only a few sites in and around a single MPA at San Miguel Island, resulting in low statistical power to resolve differences in responses at this island relative to warmer parts of the region. However, responses similar to those seen in this study at San Miguel Island were documented after five years of protection in the central California reserves established as part of the Marine Life Protection Act^64^,^65^. The central California region hosts fish communities and physical conditions similar to those at San Miguel Island, supporting the conjecture that these conditions may be associated with slower responses to marine protection.

## Conclusion

### A conceptual model of responses to marine reserve protection

**Figure 5:**
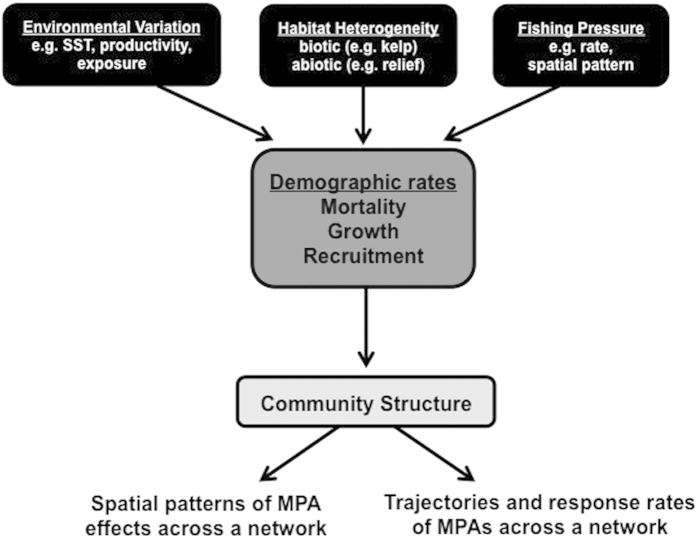
Conceptual model of effects of various biotic, abiotic and anthropogenic parameters on patterns of MPA responses across space and through time.

Environmental variation, habitat heterogeneity (abiotic and biotic), and fishing pressure will all affect the spatial and temporal patterns of responses of organisms to the establishment of marine reserves, and these responses may be observed both inside and outside of protected areas (Fig. 5). Environmental conditions (e.g. SST, productivity) have strong effects on recruitment into populations^63^,^66^,^67^ as well as other demographic rates (e.g. growth, mortality)^68^. Habitat characteristics can also affect species composition indirectly, for example through natural mortality rates (e.g., provision of shelter may alter mortality rates) or directly through habitat choice. Finally, the degree of fishing pressure, which is often related to fisheries management policies outside of MPAs, will of course be a major determinant of response to protection for fished species. Ultimately, all of these factors can act on demographic rates such as mortality, recruitment, and growth, which in turn may dictate the pace of response to protection in MPAs as well as the spatial patterns across a network.

Early assessments of marine reserves simply measured differences inside vs. outside of MPAs, with little ability to distinguish between the effects of protection and other sources of spatial variability, and they lacked consideration of the effects that reserves can have on nearby areas not currently under protection. Presently, there is much more attention given to statistical design, the effects of variation in the habitat or other environmental parameters, and the effect of differential fishing, though rarely are all measured in a single study. While assessments of single small reserves may be less prone to biases due to reserve placement and habitat differences between reserve and ‘control’ areas, the trend more recently has been to implement networks or large reserves across extensive areas that can encompass gradients of environmental conditions and fishing pressure. Assessment across these gradients is more challenging, and the effects of variation in the parameters shown in Fig. 5 are likely to affect measurements of success. Here, we demonstrate that explicitly taking account of changes through time and of differences across space lends insight to the potential for reserve protection to achieve its goals.

## Methods

### Location

The study took place in the Northern Channel Islands in California, the site of the Channel Islands National Marine Sanctuary and Channel Islands National Park (Fig. 1). In 2003, a network of Marine Protected Areas was implemented across the islands (including the more isolated Santa Barbara Island, which was omitted from these analyses due to infrequent sampling). This area is a biogeographic transition zone where the colder equatorward-flowing California current meets the warmer poleward-flowing southern California countercurrent^41^. The four islands span a large gradient in environmental conditions, including sea surface temperature and productivity^41^, and demographic measures such as recruitment^63^,^69^,^70^ and growth rates^68^,^71^,^72^. This gradient ultimately leads to different marine communities across the island chain in the rocky intertidal^42^ and on nearshore subtidal reefs^9^. Rocky reef communities on the easternmost Anacapa Island and eastern part of Santa Cruz Island are characterized by subtropical and warm temperate families^9^,^73^,^74^. Moving west towards San Miguel Island, these families are replaced by cold-temperate groups.

### Dive surveys

Fish assemblages were surveyed annually as part of a long-term monitoring program conducted by the Partnership for Interdisciplinary Studies of Coastal Oceans (PISCO) using standard underwater visual belt survey methods (www.piscoweb.org^9^). We analyzed data from 47 PISCO sites at the northern Channel Islands that were sampled annually from at least 2003 to 2012. We also excluded 3 sites on Anacapa where a much older MPA had already been established in 1978. MPAs on each island were sampled annually during June-October and we surveyed multiple sites inside and outside of any individual MPA. Details of MPA characteristics such as size and coastline extent are given in Hamilton *et al.*^9^. At each site, we conducted 8 to 12 fish transects that measured 30 × 2 × 2 m at multiple levels in the water column: benthic, midwater, and kelp canopy (when present). Transects are laid out in a stratified random design, with multiple nonpermanent transects located in fixed strata (i.e., outer, middle, and inner edges of the reef). At each level in the water column, one SCUBA diver per transect counted and estimated the sizes of all fish to the nearest centimeter (total length), excluding small cryptic fishes.

Length estimates of fishes from visual censuses were converted to weights based on allometric relationships found in published^75^ and web based (www.fishbase.org) sources. In the cases where length-weight information did not exist for a given species, the parameters from similar-bodied congeners were used. All biomass estimates were converted to metric tons per hectare (t ha^−1^) to facilitate comparisons with other studies in California and more globally. For each fish species, we summed biomass over the different levels in the water column and calculated the mean biomass per site per year (averaging across all transects at the site). These estimates represented the lowest level of replication for all analyses.

### Environmental drivers

To test the relative strength of using environmental proxies versus the island-level blocking term, we assessed the thermal environment at sites using 15-d composite sea surface temperatures (SST) created from MODIST (2000-present) and MODISA (2002-present) with 1km resolution (http://spg.ucsd.edu/Satellite_Data/California_Current/). For each survey site, we used the mean SST in a 10 × 10-km rectangle centered as closely on the site as grid resolution allowed. The 10 × 10-km average was found to be more stable and to provide a more continuous time series than resulted if only the single pixel closest to the site was used.

### Statistical analysis

To compare current spatial patterns in targeted and non-targeted fish biomass to those reported previously^9^, we calculated the average biomass for each group at each site across all years sampled. Species were categorized and then grouped as targeted or non-targeted by commercial and/or recreational fisheries by examining the California Department of Fish and Wildlife landings records from areas in the northern Channel Islands and consulting with fisheries managers and fishermen (Table S2). The effect of protection and geography on total mean biomass of each group was tested with ANOVA, treating MPA status and island as categorical fixed effects.

To evaluate whether changes in fish biomass differed between the MPA and non-MPA sites following MPA establishment, we used general linear models to test for the effects of protection (i.e. MPA status) and time, as well as an interaction between the two. MPA status was categorical and year was treated as continuous (similar to an ANCOVA model). We were particularly interested in a potential interaction between year and MPA status, which would indicate that the rate of change in biomass was different in protected and unprotected areas. Separate models were run for targeted and non-targeted species groupings to account for the direct and indirect effects of fishing. We evaluated two separate linear models accounting for biogeographic variation in different ways, either by adding a categorical biogeographic blocking variable based on the island (following^9^), or a continuous environmental variable (mean sea surface temperature, described above). We tested the statistical fit of these models using Aikake’s information criteria with a correction for small sample size (AICc) following recommendations in^76^. Statistical significance was determined in cases where the difference in AICc values (ΔAICc) was ≥2. All biomass data were log (N + 0.1) transformed prior to analysis to obtain normality. Each model was initially evaluated including all possible interactions, with non-significant interactions (all with p-values above 0.15) being dropped in the final reduced models, with the exception of the interaction between year and MPA status (see^77^). The year-by-MPA interaction was retained in all models because it was the specific hypothesis being tested, that the rate of change over time (i.e., slope of the regression model) is different in protected and unprotected areas.

In a separate model, we also compared species biomass trajectories outside of MPAs, searching for evidence of network level effects on non-MPA fish communities. We used a linear model to test the effect of targeted status, island, and year on log (N + 0.1) transformed biomass. Targeted status and island were treated as categorical variables and year treated as a continuous variable. As with the previous analysis, all interactions were evaluated and non-significant interactions dropped in the reduced model presented here.

To explore in detail the spatial variation in the rate of change in biomass of targeted and non-targeted fish since the establishment of MPAs, we used linear regression to calculate the slope of the relationship between fish biomass and time at each individual site in the dataset. To test whether the mean rate of change for each fish biomass group was increasing (positive) or decreasing (negative) at sites inside and outside of MPAs, we compared the distribution of the site-specific slopes to the null hypothesis of no change (slope = 0) using 1-sample t-tests.

To explore responses of individual species over time, we examined the biomass density of each of the top 28 most abundant individual fish species or taxonomic groupings (where species cannot be identified) in the dataset. For each of these species we ran a general linear model in which its observed biomass was predicted with year (a continuous variable) and a biogeographic variable (either the categorical variable Island, Supplementary Table S2A; or the continuous variable SST, Supplementary Table S2B). Abundance was log (N + 0.1) transformed prior to analysis to achieve normality of residuals. Sites inside MPAs were analyzed separately from those outside MPAs, because we were interested both in whether changes in abundance were occurring inside protected areas (potentially due to absence of fishing) and whether changes were occurring outside of protected areas (potentially due to spillover or displacement of fishing effort). Because of the large number of tests being performed here, we corrected significance levels based on the linear step-up methods that control the false discovery rate (proc multtest; FDR)^78^. All statistics were done using SAS (SAS Institute) and R (R Core Team 2013).

## Additional Information

**How to cite this article**: Caselle, J. E. *et al.* Recovery trajectories of kelp forest animals are rapid yet spatially variable across a network of temperate marine protected areas. *Sci. Rep.* **5**, 14102; doi: 10.1038/srep14102 (2015).

## Supplementary Material

Supplementary Tables

## Figures and Tables

**Table 1:** Results of general linear models testing the effect of protection (i.e., MPA status), geography (island) and the interaction between the two for A) average biomass of targeted species and B) average biomass of non-targeted species over period 2003–2012 Biomass data were log (N + 0.1) transformed prior to analysis.

Source	DF	Type IIISS	MeanSquare	FValue	Pr > F
**A**					
Island	3	3.0175	1.0058	4.29	0.0104
MPA	1	3.8244	3.8244	16.30	0.0002
MPA* Island	3	0.7307	0.2436	1.04	0.3865
**B**					
Island	3	2.6470	0.8823	4.97	0.0052
MPA	1	0.5368	0.5368	3.02	0.0901
MPA* Island	3	1.0392	0.3464	1.95	0.1375

**Table 2:** Results of general linear models testing the effect of protection (i.e., MPA status), geography (island), and year for A) biomass of targeted species and B) biomass of non-targeted species.

Source	DF	Type III SS	F Value	Pr > F	
**A**					
Island	3	19.6265	21.37	<0.0001	
MPA	1	5.2392	17.12	<0.0001	
Year	1	27.2279	88.95	<0.0001	
Year*MPA	1	5.2708	17.22	<0.0001	
**B**					
Island	3	22.1155	28.01	<0.0001	
MPA	1	0.3997	1.52	0.2184	
Year	1	1.7858	6.79	0.0095	
Year*MPA	1	0.4024	1.53	0.2169	

Biomass data were log (N + 0.1) transformed prior to analysis.

**Table 3:** Results of general linear models testing the effect of protection (i.e., MPA status), environment (Sea Surface Temperature; SST), and year for A) biomass of targeted species and B) biomass of non-targeted species.

Source	DF	Type III SS	F Value	Pr > F
**A**				
SST	1	20.8218	68.97	<0.0001
MPA	1	5.3365	17.68	<0.0001
Year	1	28.1522	93.25	<0.0001
Year*MPA	1	5.3646	17.77	<0.0001
**B**				
SST	1	11.3391	39.51	<0.0001
MPA	1	0.4032	1.40	0.2366
Year	1	1.2471	4.35	0.0377
Year*MPA	1	0.4061	1.42	0.2349

Biomass data were log (N + 0.1) transformed prior to analysis.
