# Supplementary material for: Recovery trajectories of kelp forest animals are rapid yet spatially variable across a network of temperate marine protected areas
Source: Sci Rep. 2015 Sep 16;5:14102. doi: 10.1038/srep14102 (PMC4642697; doi:10.1038/srep14102)
Supplement: Supplementary Tables [file srep14102-s1.pdf]

Recovery trajectories of kelp forest animals are rapid yet spatially variable across a network of temperate marine protected areas

Jennifer E. Caselle[1]*, Andrew Rassweiler[1], Scott L. Hamilton[2] and Robert R. Warner[3]

1 - Marine Science Institute, University of California, Santa Barbara, CA 93106 USA

2 - Moss Landing Marine Laboratories, 8272 Moss Landing Rd., Moss Landing, CA 95039 USA

3 - Department of Ecology, Evolution, and Marine Biology, University of California, Santa Barbara, CA 93106 USA

* corresponding author

Supplementary Figures and Tables

Supplementary Table S1. Comparison of model fits for models using the categorical variable of Island as a blocking term versus using long-term mean sea surface temperature (SST) at each site. Results of models for individual variables are shown in Table 2 and Table 3. Here we present the overall model fits for Targeted species (A) and Non-targeted species (B). In every case, biomass of the group Targeted species and Non-targeted species was log (N+0.1) transformed.

A) Targeted Species

i. Model using Island as a blocking variable

Source	DF	SS	MS	F	Pr > F
Model	6	63.8289	10.6382	34.75	<.0001
Error	428	131.0087	0.3061		
Corrected Total	434	194.8375			

R-Square	CV	Root MSE	Mean
0.328	-54.113	0.553	-1.022

ii. Model using SST as covariate

Source	DF	SS	MS	F	Pr > F
Model	4	65.0241	16.2560	53.85	<.0001
Error	430	129.8134	0.3019		
Corrected Total	434	194.8375			

R-Square	CV	Root MSE	Mean
0.334	-53.741	0.550	-1.022

B) Non-targeted Species

i. Model using Island as a blocking variable

Source	DF	SS	MS	F	Pr > F
Model	6	28.6509	4.7752	18.15	<.0001
Error	428	112.6243	0.2631		
Corrected Total	434	141.2752			

R-Square	CV	Root MSE	Mean
0.203	-86.391	0.513	-0.594

ii. Model using SST as covariate

Source	DF	SS	MS	F	Pr > F
Model	4	17.8745	4.46865	15.57	<.0001
Error	430	123.4008	0.2870		
Corrected Total	434	141.2752			

R-Square	CV	Root MSE	Mean
0.127	-90.219	0.536	-0.594

Supplement Table S2. Results of general linear models testing slopes of individual species biomass (square-root-transformed). A) Model results using Island as blocking term. B) Model results using SST as blocking term. See text for details. Species presented here are those that were most abundant in the dataset and mean biomass density (metric tons/ha) across the study region is presented. Grey cells indicate significant negative, or declining biomass while blue cells indicate significant positive or increasing biomass over time.

Species	Target Status	Mean Biomass Density	A. Island		B. SST	
			IN MPA	OUT MPA	IN MPA	OUT MPA
Blacksmith	Non-target	0.074	+	+		
Garibaldi	Non-target	0.045				
Giant kelpfish	Non-target	0.0004				
Halfmoon	Non-target	0.023	-		-	
Juvenile rockfish	Non-target	0.001				
Kelp surfperch	Non-target	0.007		- - -		- - -
Opaleye	Non-target	0.106				
Painted greenling	Non-target	0.005	+++	+++	+++	+++
Rainbow surfperch	Non-target	0.002				
Rock wrasse	Non-target	0.005		++		+
Señorita	Non-target	0.052		+		+
Shiner perch	Non-target	0.0002				
Silversides	Non-target	0.0001				
Black & Yellow rockfish	Target	0.004	+		+	+
Black surfperch	Target	0.048				
Blue rockfish	Target	0.046				
Cabezon	Target	0.004	++	+++	++	+++
CA sheephead	Target	0.121	+++		+++	
Copper rockfish	Target	0.007				
Gopher rockfish	Target	0.003	+		+	
Kelp bass	Target	0.141	+++		+++	
Kelp rockfish	Target	0.055	+++	++	+++	++
Lingcod	Target	0.012	+		+	
Ocean whitefish	Target	0.013				
Olive rockfish	Target	0.019				
Pile surfperch	Target	0.027				
Rubberlip surfperch	Target	0.024	+		+	
Striped surfperch	Target	0.035	+		++	+

+ or - $p<0.05$, ++ or - - $p<0.01$, +++ or - - - $p<0.001$ after adjusting p-values to control the false discovery rate.
